# P04.62. Systematic review of clinical studies of whole practice naturopathic medicine

**DOI:** 10.1186/1472-6882-12-S1-P332

**Published:** 2012-06-12

**Authors:** C Calabrese, E Oberg, R Bradley, D Seely, K Cooley, J Goldenberg

**Affiliations:** 1 Naturopathic Physicians Research Institute, Portland, USA; 2 Bastyr University, Kenmore, USA; 3 Canadian College of Naturopathic Medicine, Toronto, Canada

## Purpose

Individualized combinations of therapeutic modalities and remedies are generally the rule in naturopathic practice with selection determined by the system’s principles and guidelines. With the wide variation in real-world use, evaluating the whole practice best assesses overall benefits and risks. Naturopathic doctors in Canada and the US in licensed jurisdictions receive accredited training to a common standard resulting in practice that may be distinct from that in unregulated jurisdictions or in other countries. We seek to represent the landscape of clinical studies in licensed North American naturopathic medicine to identify gaps in knowledge and generate hypotheses for future study.

## Methods

Through a systematic review, we accessed clinical studies in acute and chronic diseases in which licensed naturopathic clinicians were allowed access to therapeutic and diagnostic tools within their scope of practice or to well-described models of current whole practice. Databases searched include AMED, EMBASE, MEDLINE, PREMEDLINE and the Cochrane Library. In addition, content experts were consulted and the lay literature hand-searched to identify additional relevant studies. The review was performed and reported according to PRISMA guidelines for systematic reviews and describes for each study participants, interventions, comparisons, outcomes, and study design.

## Results

We have so far identified 12 studies fitting inclusion criteria with a variety of designs in anxiety, tendinitis, temporomandibular joint disorder, low back pain, general pain, hypertension, multiple sclerosis, menopausal symptoms, cardiovascular risk and type 2 diabetes. Six were randomized trials including 2 with cost components, one a comparative prospective observational study, one a prospective single group observation, and four were retrospective. All showed some evidence of effectiveness though most had methodological weaknesses. No studies in acute disease meeting criteria were found.

## Conclusion

The review provides evidence of effectiveness and cost savings that merit further investigation of naturopathic care for chronic disease.

